# Observations from the RESET clinical trial: A post-hoc per protocol analysis of clinical outcomes with percutaneous 60-day medial branch PNS in chronic low back pain

**DOI:** 10.1016/j.inpm.2026.100775

**Published:** 2026-06-06

**Authors:** Sean Li, Zachary L. McCormick, Denise D. Lester, Michael J. DePalma, Christopher A. Gilmore, Jessica B. Jameson, Mehul J. Desai, Tristan E. Weaver, Shivanand P. Lad, Scott J. Davidoff, Drew M. Trainor, Kasra Amirdelfan, Mitchell P. Engle, Timothy R. Deer, Thomas S. Lee, Francesco Vetri, Meenakshi Bindal, Melissa A. Tornero-Bold, Morad N. Nasseri, Steven P. Cohen, William H. Clark, Meredith J. McGee, Joseph W. Boggs

**Affiliations:** a Premier Pain Centers, Shrewsbury, NJ, USA; b University of Utah School of Medicine, Salt Lake City, UT, USA; c Central Virginia VA Health System, Richmond, VA, USA; d Virginia iSpine Physicians, Richmond, VA, USA; e Center for Clinical Research, Winston Salem, NC, USA; f Axis Spine Center, Coeur d’Alene, ID, USA; g International Spine, Pain & Performance Center, Washington, DC, USA; h The Ohio State University, Columbus, OH, USA; i Duke University, Durham, NC, USA; j Main Line Spine, King of Prussia, PA, USA; k Denver Spine & Pain Institute, Greenwood Village, CO, USA; l Boomerang Healthcare, Walnut Creek, CA, USA; m Institute of Precision Pain Medicine, Corpus Christi, TX, USA; n The Spine and Nerve Center of the Virginias, Charleston, WV, USA; o Rehabilitation Institute at Sinai, LifeBridge Health, Baltimore, MD, USA; p Millennium Pain Center, Bloomington, IL, USA; q Northwestern University, Chicago, IL, USA; r Walter Reed National Military Medical Center, Bethesda, MD, USA; s SPR, Cleveland, OH, USA

**Keywords:** Pain management, Back pain, Chronic pain, Low back pain, Medial branch, Medial branch block, Facetogenic, Discogenic, Discovertebral, Clinical features

## Abstract

**Background:**

Percutaneous 60-day peripheral nerve stimulation (PNS) of the lumbar medial branches is a minimally invasive treatment option for chronic low back pain (CLBP). A recent randomized controlled trial (RESET Clinical Trial) met its primary endpoint, with a significantly greater proportion of participants treated with 60-day PNS (n = 112) reporting ≥50% reductions in average low back pain compared to those treated with physician-directed usual care with standard interventional management (n = 110; e.g., ablations, injections, physical therapy) at 3 months post start of treatment.

**Objective:**

Building on the published findings from the Full Analysis Set, this post-hoc analysis primarily explored whether per protocol treatment delivery (bilateral stimulation that generated sensations covering the majority of the painful region in the low back for 6-12 h per day) was associated with response to 60-day PNS. Additional post-hoc evaluations explored the influence of clinical variables across the study population and investigated outcomes within exploratory subgroups of common clinical pain profiles (e.g.*,* patients with characteristics typical of facetogenic and discovertebral pain).

**Methods:**

Univariate logistic regressions evaluated associations between variables (treatment delivery and clinical variables) and responder rate (≥50% reduction in average pain at 3 months).

**Results:**

The results suggest per protocol treatment delivery is associated with positive outcomes following treatment with 60-day PNS for CLBP (odds ratio = 3.04, 95% confidence interval = [1.16, 7.93], p = 0.023). Within this clinical trial's population as defined by the inclusion and exclusion criteria, neither clinical variables (e.g., age, pain duration, gender, average pain intensity, pain interference, and disability) nor response to a medial branch block had a significant impact on treatment response. Additional post-hoc analyses showed positive outcomes with 60-day PNS among exploratory cohorts defined by common facetogenic and discovertebral pain features. Reductions in pain extended to improvements in pain interference and disability, with benefits sustained at all completed timepoints through 6 months (follow-up beyond 6 months is ongoing).

**Conclusions:**

Together, these findings suggest that 60-day PNS treatment delivery is an important determinant of outcomes, underscoring the role of proper lead implantation, patient education, and treatment adherence in clinical practice.

**ClinicalTrials gov identifier:**

NCT04246281.

## 1 Introduction

Chronic low back pain (CLBP) affects over 10% of the U.S. population annually [1,2], with the average sufferer reporting significant disability, interference with activities of daily living, and substantial reductions in quality of life [[3], [4], [5]]. While underlying features of CLBP can be difficult to isolate and classification remains an area of active debate [6], understanding what drives treatment response, including the role of treatment delivery (e.g., encompassing both clinical execution and patient adherence), is an area of growing clinical interest. Among available interventions for CLBP, percutaneous 60-day peripheral nerve stimulation (PNS) is a minimally invasive neuromodulation treatment that can provide pain relief extending beyond the short-term treatment period, supported by prospective clinical trials and real-world data across various pain conditions (e.g., Refs. [[7], [8], [9]]).

A recent multicenter randomized controlled trial (RCT) involving over 200 participants, the RESET Clinical Trial, met its primary clinical endpoint in the study's Full Analysis Set, with a significantly greater proportion of participants treated with 60-day PNS (n = 112) reporting ≥50% reductions in average low back pain compared to those treated with physician-directed usual care with standard interventional management (n = 110; e.g., physical therapy, injections, ablations; p < 0.001) at 3 months post start of treatment [10]. Percutaneous 60-day PNS is a time-limited treatment intervention in which stimulation delivery may meaningfully contribute to treatment response, though this relationship has not been thoroughly explored. This report details a post-hoc analysis of the RESET Clinical Trial evaluating the relationship between treatment delivery and outcomes with 60-day PNS. The trial enrolled a carefully screened cohort of adults with chronic, moderate-to-severe refractory low back pain who had failed multiple prior therapies, while excluding individuals with radicular symptoms, pain from the sacroiliac joint, greater than mild lumbar scoliosis, clinically relevant spinal stenosis, or prior lumbar surgery. Notably, this trial did not require specific etiological diagnoses for enrollment and included a range of CLBP presentations, including patients with features of two of the most common types of axial back pain, facetogenic and discovertebral pain [11,12], underscoring the need for continued exploration.

The main objective of this post-hoc analysis was to test the hypothesis that per protocol treatment delivery (prospectively defined as bilateral stimulation that generated sensations covering the majority of the painful region in the low back for 6-12 h per day) would be associated with positive treatment response at the study's primary endpoint. Additional objectives of this analysis evaluated whether clinical variables were associated with success and explored outcomes of 60-day PNS within the defined study population among patients with common CLBP features, such as those consistent with facetogenic pain and discovertebral pain.

## 2 Materials and methods

Full methods from the RESET Clinical Trial have been previously published [10]. This report is a post-hoc analysis of the 60-day PNS group; key methodological elements relevant to the analysis and textual material from the prior publication are included when appropriate for context. This study received its initial institutional review board (IRB) approval from a central IRB (WIRB, now WCG IRB, Princeton, NJ) on December 30, 2019, and was registered on ClinicalTrials.gov (NCT04246281) on January 29, 2020. Funding for this study was received from the Department of Defense (see Funding Statement). The protocol is available on ClinicalTrials.gov and all methods and procedures followed the principles of the Declaration of Helsinki. All participants in the RESET Study presented with CLBP without significant radiation to the lower extremities and provided written, informed consent prior to completion of any study procedures.

### 2.1 Eligibility criteria & start of treatment

Participants were required to have moderate to severe refractory CLBP lasting >6 months, with pain primarily in the lumbar region and an average pain intensity (BPI5) score of ≥4 on a 0–10 scale. Key inclusion criteria included prior use of at least two types of pain therapies (e.g., physical therapy, pharmacologic management, injections), active health insurance, and stable treatment for the previous 4 weeks. Key exclusion criteria included radicular or referred pain outside the lumbar region with pain score ≥4, BMI >40, prior lumbar radiofrequency ablation within 6 months, prior spine surgery, significant spinal abnormalities (e.g., greater than mild scoliosis, clinically spinal relevant stenosis), or contraindications to the percutaneous 60-day PNS device (e.g., pregnancy, implanted devices). Participants with a BPI9 pain interference score <4, BDI-II score >20, and who reported taking ≥90 MME of opioids at baseline were also excluded. A specific etiological diagnosis was not required for enrollment and patients with a range of presentations (including the two most common types of axial CLBP: facetogenic and discovertebral pain [11,12]) were enrolled, as the other most common types of CLBP were excluded via the eligibility criteria (e.g., sacroiliac pain, radicular pain, persistent spinal pain syndrome, symptomatic spinal stenosis, etc.). A full list of eligibility criteria is shown in **appendix 1.** Eligibility was confirmed based on a physical exam, CLBP history, and completion of a 7-day pain diary (BPI5) with a required mean score ≥4.

Participants were randomized 1:1 to either Group #1 (Percutaneous 60-Day PNS) or Group #2 (physician-directed usual care with standard interventional management). The “start of treatment” (SOT) was defined as the initial date of the intervention (e.g.*,* date of Percutaneous PNS lead implantation in Group #1). This report focuses on Group #1 to explore associations between per protocol treatment delivery and response to 60-day PNS, with secondary objectives of evaluating the effect of clinical variables and exploring outcomes in patients with common CLBP features.

### 2.2 Medial branch block (MBB)

Group #1 participants without a documented record of a previous MBB that occurred within 3 months of enrolling in the study proceeded to receive a MBB. This MBB was included in the protocol to support an exploratory analysis of its prognostic value for identifying percutaneous 60-day PNS responders. Prior to the start of the MBB procedure, participants reported their current low back pain score on a scale of 0 (no pain) to 10 (worst pain imaginable). Physicians performed an anesthetic nerve block of the medial branch of the dorsal ramus using a standardized procedure including 0.5 mL of 1% lidocaine at each of 2-3 lumbar levels centered around the region of pain, without steroids or other anesthetic compounds. Participants were then asked to report their low back pain score immediately following the block and completed a MBB diary recording their low back pain at 1- and 2-h post MBB. The present analysis considered the response to the MBB procedure to be positive if the participant reported ≥80% reduction in pain lasting ≥1 h post MBB [13]. If a MBB diary was not available, the diagnostic response was determined based on their immediate post-MBB score. The results presented here are based on a single block, whereas dual blocks are commonly used in clinical practice.

### 2.3 Percutaneous 60-day PNS lead implantation

At least 1 week after the MBB, Group #1 participants received the SPRINT® Peripheral Nerve Stimulation (PNS) System (SPR® Therapeutics, Inc. (“SPR”), Cleveland, OH), an FDA 510(k) cleared (approved) device used on-label for the treatment of chronic pain in the low back. Bilateral percutaneous MicroLead™ open-coil PNS leads (SPR) designed to be placed remote from the nerve [14,15] were implanted under ultrasound and/or fluoroscopic image guidance to target the lumbar medial branches of the dorsal ramus nerves in the center of the level of pain [10]. Successful lead implantation targeting the medial branch nerves was confirmed by visualization of stimulation-evoked multifidus muscle tension with ultrasound imaging and by participant-reported comfortable sensations overlapping the painful region. A small external pulse generator was connected to the percutaneous leads and delivered stimulation that generated comfortable sensations in the region of pain (stimulation parameters: frequency: 12 Hz; duty cycle: 50%; amplitude range: 0–30 mA; pulse duration range: 10–200 μs).

### 2.4 Per protocol treatment delivery of percutaneous 60-day PNS

Participants were instructed to use the 60-day PNS device for 6-12 h per day at an intensity level that produced strong, but comfortable sensations covering the majority of their painful low back region, while maintaining their usual activities. Throughout the study, trained study personnel (e.g., physician, nurse, or study coordinator) interrogated each participant's device to assess treatment adherence and provided participants with a body diagram to facilitate assessment of the location of pain and stimulation sensations. Participants reported a value (0-100%) for the amount of the painful region that was covered by stimulation-evoked sensations. According to the prospectively-defined statistical analysis plan, participants were included in the Per Protocol Set (*i.e.,* received the treatment as prescribed) if bilateral stimulation usage totaled ≥336 h (6 h per day for 56 days [8 weeks]) and consistently provided stimulation sensations overlapping >50% of the painful area throughout treatment (assessed at lead implant, 1-month visit, and end of treatment).

### 2.5 Chronic low back pain features

In this post-hoc analysis, response to 60-day PNS was explored in relation to potential features of facetogenic or discovertebral pain, the two most common types of axial CLBP within the RESET study's inclusion and exclusion criteria. It is important to note that there are many clinical diagnostic tests (e.g., provocative discography, dual diagnostic facet joint blocks) that may be more sensitive for definitively identifying certain CLBP etiologies [11]. This analysis pragmatically explored the available data collected during the clinical study to identify participants with potential features of facetogenic or discovertebral pain; however, these features do not establish definitive diagnoses.

Information regarding each participant's low back pain history was initially recorded at baseline. Each participant's low back pain features were categorized as likely components of facetogenic (characterization f_1_: “patient history”), discovertebral (characterization d_1_: “patient history”), or other (i.e., participants who were not characterized to have dominant facetogenic or discovertebral pain features). To develop more objective characterizations of facetogenic and discovertebral pain features for the present analysis, baseline characteristics, pain during physical examination provocative maneuvers, and results from diagnostic testing or imaging collected prior to the start of treatment were used.

Facetogenic pain features were explored as being characterized using two criteria: i) via a clinical examination that revealed localized paraspinal tenderness during palpation and pain during prolonged standing (characterization f_2_) [16,17], and ii) via a positive response to a MBB (characterization f_3_). The primary facetogenic cohort of interest was defined as participants meeting either or both criteria (characterization f_4_). Findings from medical imaging (e.g., lumbar spine radiographs, MRI, CT scans) were systematically checked for keywords that may be associated with discovertebral pain (e.g., moderate or severe disc degeneration, bulge, protrusion, height loss, herniation, Type 1 and/or Type 2 Modic changes, or annular fissures). Discovertebral pain features were explored as being characterized using two criteria: i) via a clinical examination that revealed pain during forward flexion and prolonged sitting (characterization d_2_) [18,19], and ii) via moderate to severe findings on medical imaging (characterization d_3_). The primary discovertebral cohort of interest was characterized as participants meeting either or both criteria (characterization d_4_).

### 2.6 Outcome measures & statistical methods

Participants recorded daily average pain intensity (BPI5) scores in weekly diaries for each follow-up study visit. The study's Primary Endpoint was predefined as the proportion of participants who experienced clinically substantial reductions in CLBP, as determined by ≥ 50% reduction in average pain intensity (BPI5) at 3 months post-treatment compared to baseline. Secondary endpoints were assessed using validated survey instruments completed at baseline and during follow-up visits. The secondary endpoints included pain interference on activities of daily living (Brief Pain Inventory, Question #9; BPI9) and back-pain related disability (Oswestry Disability Index; ODI). Data collection, monitoring, and analysis are complete through 6-months post-treatment.

The statistical analysis plan prespecified post-hoc evaluations of the relationship between clinical variables and success for the primary endpoint, determining their influence on the likelihood of achieving ≥50% reduction in average pain with 60-day PNS at 3 months. It is important to note that the study population was defined by inclusion and exclusion criteria that bounded the range of certain clinical variables. These criteria were informed by prior percutaneous 60-day PNS studies [[20], [21], [22]]. For example, participants must have had a BMI <40 and opioid consumption <90 mg morphine equivalents (MME) at baseline, which restricted the observed variability in these measures and limited the ability to assess their full influence on treatment outcomes. As such, these variables were not included in the present analysis. The variables explored in this report included “Per Protocol Treatment Delivery”, participant demographics and clinical characteristics (“Age”, “Chronic Low Back Pain Duration”, and “Gender”), baseline scores (“Average Pain Intensity, BPI5”, “Pain Interference, BPI9”, “Oswestry Disability Index, ODI”), and diagnostic tests (“≥80% Relief from a Single MBB”).

For each variable, univariate logistic regressions were performed to estimate odds ratios (ORs) with 95% Wald confidence limits and Chi-Square p-values, which reflect the direction and magnitude of effect on treatment response (OR >1 represents increased odds of a positive treatment response, OR <1 signifies decreased odds). Continuous variables were standardized to mean 0 and standard deviation (SD) 1; ORs for continuous variables reflect the change per 1-unit increase in the original scale. To adjust for potential confounders, multivariate logistic regression models were conducted as sensitivity analyses. Full models including the assessed variables were fit for the Full Analysis Set (“Per Protocol Treatment Delivery”, “Age”, “Chronic Low Back Pain Duration”, “Gender”, “Average Pain Intensity, BPI5”, “Pain Interference, BPI9”, “Oswestry Disability Index, ODI”, and “≥80% Relief from a Single MBB”) and the Per Protocol Set (“Age”, “Chronic Low Back Pain Duration”, “Gender”, “Average Pain Intensity, BPI5”, “Pain Interference, BPI9”, “Oswestry Disability Index, ODI”, and “≥80% Relief from a Single MBB”). For all statistical tests, a two-sided alpha of 0.05 was applied. Success rates, defined as the proportion of participants achieving ≥50% reduction in average pain, were reported for clinically relevant cohorts. Data are presented as mean ± standard deviation unless otherwise noted.

## 3 Results

### 3.1 Study population

As reported in McCormick et al. [10], 103 participants were randomized to and received percutaneous 60-day PNS (9 participants from the 112 randomized to Group #1 withdrew their study participation prior to receiving 60-day PNS). A total of 93 participants completed the end of treatment visit at 2 months, 91 participants completed the Primary Endpoint visit at 3 months, and 89 participants completed the 6-month follow-up visit. A CONSORT flow diagram is provided in the Full Analysis report [10]. Baseline demographics for participants in the Full Analysis Set are reported in Table 1. All study adverse events were non-serious (mild or moderate) and were followed to resolution, as reported previously [10]. Of the 93 participants who completed the end of treatment visit, 72% (n = 67) completed the treatment as prescribed (representing 60% of the 112 randomized participants and 65% of the 103 participants who started treatment). No participant received bilateral stimulation totaling ≥720 h (12 h per day for 60 days). Of the 91 participants who completed the primary endpoint visit at 3 months, 83 received an MBB as part of the study protocol and 8 had a documented MBB within 3 months of enrollment. MBB diaries were available for 71 of 83 participants (86%) who received a study MBB; for the remaining 12, diagnostic response was determined from the immediate post-MBB pain score.

**T A B L E 1:** Participant characteristics of 60-day PNS cohort (group #1).

	**Full Analysis Set (n=112)**
***Participant demographic and clinical characteristics***	
Age (years), mean ± SD	55 ± 14
CLBP duration (years), mean ± SD	14 ± 13
Female, % (n)	53% (59)
***Baseline scores***	
Average pain intensity, BPI5, mean ± SD	6.0 ± 1.2
Pain interference, BPI9, mean ± SD	6.2 ± 1.4
Oswestry Disability Index (% Point), ODI, mean ± SD	42 ± 16
***Per protocol treatment delivery (among n = 93 participants who completed treatment)***	
Per protocol: completed treatment as prescribed, % (n)	72% (67)

### 3.2 Assessed clinical variables

As shown in Table 2, among the overall Group #1 population (Full Analysis Set), per protocol treatment delivery of percutaneous 60-day PNS (6-12 h of stimulation per day and sensations covering the majority of the painful region of the low back) was significantly associated with achieving ≥50% reduction in average pain at 3 months (odds ratio = 3.04, 95% confidence interval = [1.16, 7.93], p = 0.023). The results from the univariate logistic regressions of other clinical variables in the Full Analysis Set suggest age, CLBP duration, gender, average pain intensity (BPI5), pain interference (BPI9), disability (ODI), and response to a MBB did not have a significant impact on treatment response within the patient population defined by the study's inclusion and exclusion criteria (p-values >0.05). When the analysis was repeated among the Per Protocol Set to control for receipt of treatment delivery as prescribed during the short-term treatment, the same clinical variables were not associated with achieving ≥50% reduction in average pain (p-values>0.05, Table 2). In a multivariate logistic regression model adjusting for potential confounding effects, per protocol treatment delivery remained the only statistically significant assessed variable associated with treatment response in the Full Analysis Set (OR = 3.03, 95% CI [1.06, 8.61], p = 0.0381). In a separate multivariate model evaluating only participants in the Per Protocol Set, none of the assessed variables were associated with outcomes (p-values>0.05).

**T A B L E 2:** Odds ratios for assessed Clinical variables.

	Full Analysis Set		Per Protocol Set	
	Odds Ratio [95% CI]	p-value	Odds Ratio [95% CI]	p-value
***Per protocol treatment delivery***^a^				
Per protocol: completed treatment as prescribed	3.04 [1.16, 7.93]	0.023	n/a	n/a
***Participant demographics***				
Age (years)	1.00 [0.97, 1.03]	0.934 (n.s.)	1.01 [0.97, 1.05]	0.716 (n.s.)
Chronic low back pain duration (years)	1.00 [0.97, 1.04]	0.865 (n.s.)	1.00 [0.95, 1.04]	0.818 (n.s.)
Gender (female)	1.33 [0.57, 3.11]	0.503 (n.s.)	0.77 [0.27, 2.15]	0.614 (n.s.)
***Baseline scores***				
Average pain intensity, BPI5	1.09 [0.77, 1.54]	0.620 (n.s.)	1.08 [0.71, 1.64]	0.712 (n.s.)
Pain interference, BPI9	1.09 [0.80, 1.48]	0.586 (n.s.)	0.87 [0.61, 1.25]	0.456 (n.s.)
Oswestry Disability Index, ODI	1.01 [0.98, 1.04]	0.589 (n.s.)	1.01 [0.97, 1.06]	0.495 (n.s.)
***Diagnostic tests***				
≥80% relief from medial branch block	2.34 [0.97, 5.66]	0.058 (n.s.)	2.21 [0.77, 6.32]	0.139 (n.s.)

Abbreviations: Brief Pain Inventory (BPI), Non-significant (n.s.), Oswestry Disability Index (ODI).

a Per protocol treatment delivery was prospectively defined as bilateral stimulation that generated sensations covering the majority of the painful region in the low back for 6-12 h per day throughout the 60-day treatment period.

### 3.3 Primary clinical endpoint: average pain intensity

**Fig. 1:**
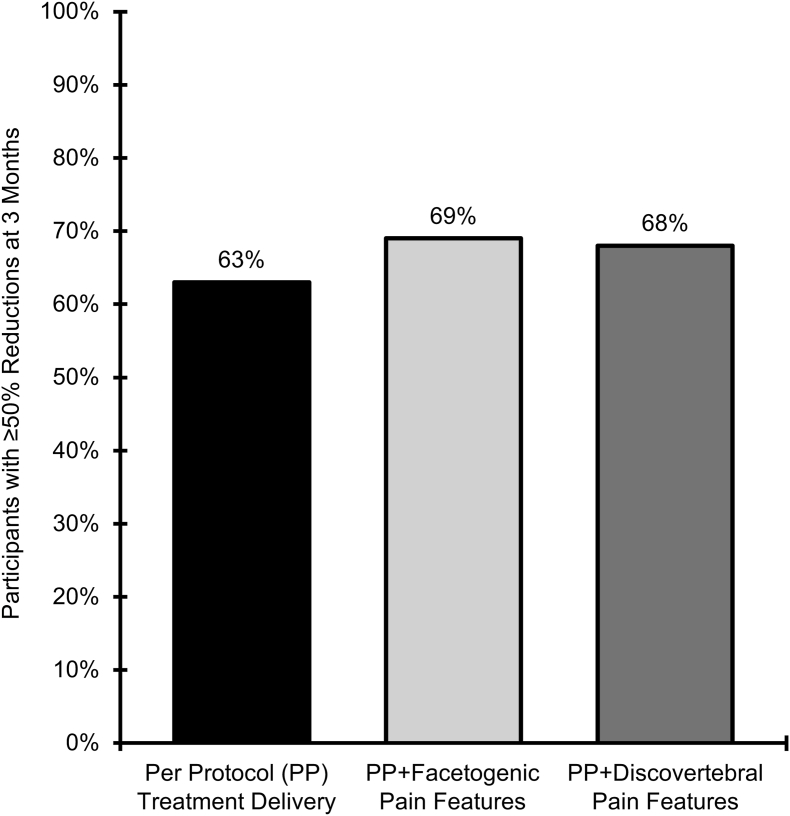
**Primary Endpoint Results: The Proportion of Participants Experiencing ≥50% Reductions in CLBP with Per Protocol Treatment Delivery of Percutaneous 60-Day PNS**. Three months post-treatment, the Primary Endpoint assessed the proportion of participants experiencing ≥50% reductions in average low back pain intensity (BPI5). Per protocol treatment delivery of 60-day PNS (bilateral stimulation that generates sensations covering the majority of the painful region in the low back for 6-12 h per day) was associated with positive outcomes for CLBP (odds ratio = 3.04, 95% confidence interval = [1.16, 7.93], p = 0.023; Table 2). Among all participants who completed the prescribed 60-day PNS treatment protocol (n = 65), 63% reported ≥50% pain relief at 3-months post start of treatment. Post-hoc assessments of exploratory cohorts within the Per Protocol set suggests 60-day PNS was effective across multiple types of axial CLBP; 69% of those with facetogenic pain features (f_4_, n = 36) and 68% of those with discovertebral pain features (d_4_, n = 40) reported ≥50% pain relief at 3 months. Abbreviations: Average Pain Intensity (BPI5), Brief Pain Inventory (BPI), Confidence Interval (CI), Peripheral Nerve Stimulation (PNS).

McCormick et al. reports on the results from the Full Analysis Set, including outcomes from the full imputed dataset (n = 112 with 60-day PNS), the observed dataset (n = 91 completed the primary clinical endpoint at 3 months), and multiple sensitivity analyses [10]. In the Full Analysis Set (full imputed dataset, n = 112), 55% reported ≥50% reduction in average pain at 3 months with 60-day PNS [10]. In this post-hoc analysis of the Per Protocol Set, as shown in Table 3 and Fig. 1, 63% of participants reported ≥50% pain relief at 3-months post start of treatment following per protocol treatment delivery (n = 65). In post-hoc assessments of exploratory cohorts within the Per Protocol Set, 69% of those with facetogenic pain features (f_4_: n = 36) and 68% of those with discovertebral pain features (d_4_: n = 40) reported ≥50% pain relief at 3 months (Table 3). Of those with unknown or ambiguous CLBP features (i.e., all other Per Protocol participants who did not meet f_4_ facetogenic or d_4_ discovertebral characterizations), 64% (n = 11) reported ≥50% pain relief at 3 months. Since there is variability in clinical approaches for characterizing pain features, the primary endpoint outcomes for multiple strategies of defining pain features are also shown in Table 3.

**T A B L E 3:** Proportion of Per Protocol participants with ≥50% reduction in average pain at 3 months.

	***All Participants, % (n)***
Per Protocol Set	63% (65)
***Facetogenic pain features, as characterized by:***	
f_1_ Patient history	63% (40)
f_2_ Pain during palpation and standing	63% (19)
f_3_ Positive MBB	72% (29)
f_4_ Pain during palpation and standing and/or positive MBB	69% (36)
***Discovertebral pain features, as characterized by:***	
d_1_ Patient history	58% (31)
d_2_ Pain during forward flexion and sitting	59% (29)
d_3_ Imaging (e.g., lumbar spine radiographs, MRI, CT scans)	61% (18)
d_4_ Pain during forward flexion and sitting and/or imaging	68% (40)

### 3.4 Secondary endpoints: additional patient-centric outcomes

**Fig. 2:**
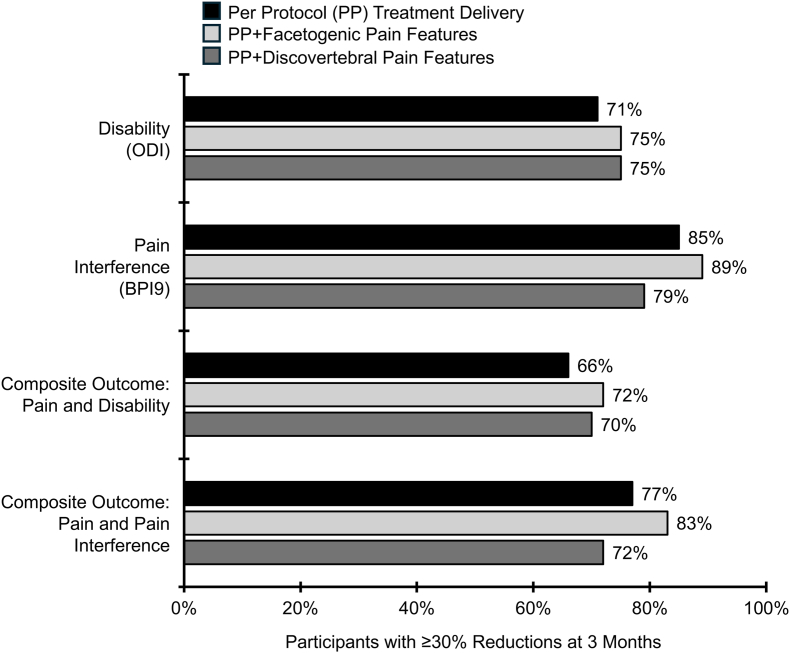
**Secondary Endpoint Results, Including Impact of Treatment on Disability, Pain Interference, and Composite Outcomes.** Results for secondary endpoints are shown at three months post-treatment. Per protocol treatment delivery of 60-day PNS for chronic low back pain was defined as bilateral stimulation that generates sensations covering the majority of the painful region in the low back for 6-12 h per day. Among all participants with per protocol treatment delivery (n = 65), reductions in pain enabled ≥30% improvements in disability (ODI; 71% with improvements), pain interference (BPI9; 85%), a composite outcome requiring improvements in both pain and disability (BPI5 and ODI; 66%), and a composite outcome requiring improvements in both pain and pain interference (BPI5 and BPI9; 77%). These results appear consistent across exploratory cohorts with facetogenic pain features (f_4_, n = 36) and discovertebral pain features (d_4_, n = 40). For those with facetogenic pain features, 75%, 89%, 72%, and 83% of participants reported ≥30% improvements in disability, pain interference, a composite outcome requiring improvements in both pain and disability, and a composite outcome requiring improvements in both pain and pain interference, respectively. For those with discovertebral pain features, 75%, 79%, 70%, and 72% of participants reported ≥30% improvements in those same endpoints. Abbreviations: Brief Pain Inventory (BPI), Oswestry Disability Index (ODI), Pain Interference (BPI9), Peripheral Nerve Stimulation (PNS).

Secondary outcomes for the Full Analysis Set were previously reported [10]. In this post-hoc analysis of 60-day PNS participants in the Per Protocol Set, as shown in Fig. 2, 71% of participants reported ≥30% improvements in disability (ODI, n = 66), 85% reported ≥30% improvements in pain interference (BPI9, n = 65), 66% reported success in a composite outcome requiring ≥30% reductions in both pain and disability (BPI5 and ODI, n = 65), and 77% reported success in a composite outcome requiring ≥30% reductions in both pain and pain interference (BPI5 and BPI9, n = 64). Secondary endpoints for those with facetogenic pain features (f_4_) and discovertebral pain features (d_4_) are shown in Fig. 2 for ≥30% improvements in disability (facetogenic: 75%, n = 36; discovertebral: 75%, n = 40), pain interference (facetogenic: 89%, n = 35; discovertebral: 79%, n = 39), a composite outcome requiring both pain and disability (facetogenic: 72%, n = 36; discovertebral: 70%, n = 40), and a composite outcome requiring both pain and pain interference (facetogenic: 83%, n = 35; discovertebral: 72%, n = 39).

### 3.5 Durability of relief

**Fig. 3:**
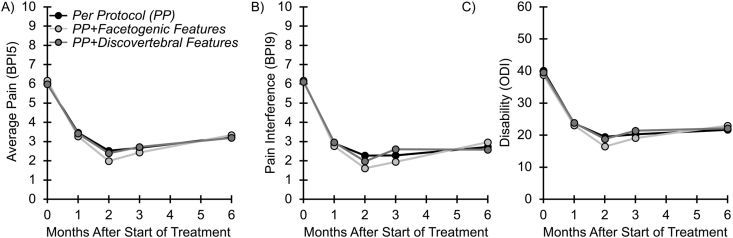
**Long-term Improvements in CLBP with Per Protocol Utilization of Percutaneous 60-Day PNS**. The mean values for all participants in the Per Protocol Set (n = 65), as well as exploratory cohorts with facetogenic pain features (f_4_, n = 36) and discovertebral pain features (d_4_, n = 40), are shown for (A) pain intensity, (B) pain interference, and (C) disability over time. Reductions in pain intensity (BPI5), pain interference (BPI9), and disability (ODI) were sustained for those with per protocol treatment delivery of 60-day PNS (bilateral stimulation that generates sensations covering the majority of the painful region in the low back for 6-12 h per day). Abbreviations: Brief Pain Inventory (BPI), Oswestry Disability Index (ODI), Pain Interference (BPI9), Peripheral Nerve Stimulation (PNS), Per Protocol (PP).

As shown in Fig. 3, the reductions in pain intensity (BPI5), pain interference (BPI9), and disability (ODI) were sustained at completed timepoints through 6 months for participants in the Per Protocol Set, as well as exploratory cohorts with facetogenic pain features (f_4_) and discovertebral pain features (d_4_). Follow-up beyond 6 months is ongoing.

## 4 Discussion

This post-hoc analysis explored the relationship between per protocol treatment delivery and treatment response in the percutaneous 60-day PNS cohort from the RESET Clinical Trial, a recent multicenter pragmatic RCT evaluating outcomes from 60-day PNS relative to physician-directed usual care with standard interventional management (published results from the Full Analysis Set are reported in McCormick et al. [10]). Secondary objectives evaluated whether clinical variables were associated with success and explored outcomes of 60-day PNS within the defined study population in patients with common CLBP features, such as those consistent with facetogenic pain and discovertebral pain. The outcomes from this follow-up analysis suggest that per protocol treatment delivery (e.g., encompassing both clinical execution and patient adherence) is associated with positive outcomes following treatment with 60-day PNS. Among those enrolled, the assessed clinical variables, including response to a diagnostic medial branch block, did not have a meaningful effect on outcomes within the study population. These results underscore the importance of proper lead implantation, patient education, and treatment adherence as key clinical and practical considerations for this short-term treatment, provided the patient's characteristics are consistent with the study's inclusion and exclusion criteria.

In this study, clinicians implanted bilateral percutaneous 60-day PNS leads targeting the lumbar medial branches of the dorsal ramus with a goal of producing comfortable sensations in the majority of the painful region and participants were instructed to use the device 6-12 h per day. This analysis found that per protocol treatment delivery was significantly associated with achieving meaningful pain relief with 60-day PNS, as those participants had 3.04 times higher odds of achieving ≥50% reduction in average pain at 3 months (Table 4, Key Insight #1). Importantly, the association between treatment delivery and response may reflect, at least in part, underlying patient, provider, or site-level factors that were not fully accounted for in the model; accordingly, the corresponding odds ratio should not be interpreted as establishing a treatment effect. Despite this, in alignment with the theorized mechanism of action of 60-day PNS, sufficient coverage and usage of stimulation may be critical in driving peripherally induced central reconditioning to provide pain relief [14]. Greater overlap of stimulation sensations with the painful region likely reflects afferent input engaging corresponding areas within the central nervous system, while adequate daily usage may produce sustained, targeted afferent input capable of providing pain relief and potentially reversing maladaptive neuroplasticity. These findings suggest proper lead implantation, including testing during implantation to verify stimulation sensations overlap the area of pain, as well as patient education, engagement, and adherence to the treatment protocol are important for increasing the likelihood of success with 60-day PNS.

**T A B L E 4:** Key insights from a per protocol analysis of percutaneous 60-day peripheral nerve stimulation (PNS) from the RESET Clinical Trial.


**Insight #1**	*Per protocol treatment delivery of 60-day PNS for chronic low back pain (bilateral stimulation that generates sensations covering the majority of the painful region in the low back for 6-12 h* per *day) is associated with a greater likelihood of achieving significant pain relief.*
**Insight #2**	*A positive medial branch block (MBB) was not statistically associated with 60-day PNS treatment response, suggesting that a positive MBB may not be necessary to identify candidates that are likely to benefit from 60-day PNS for chronic low back pain (CLBP)*.
**Insight #3**	*Post-hoc assessments suggest positive treatment outcomes in exploratory cohorts of patients with facetogenic or discovertebral (including discogenic) pain features following treatment with 60-day PNS for CLBP*.

Within the patient population defined by the study's inclusion and exclusion criteria, participant demographics and clinical characteristics (age, chronic low back pain duration, gender), baseline scores (pain, pain interference, disability), and diagnostic tests (≥80% relief from a single MBB) did not significantly influence the likelihood that participants would achieve ≥50% reduction in average pain at 3 months. In this study, sensitivity analyses were conducted using multivariate logistic regression models to adjust for potential confounding effects. Across all modeling approaches, per protocol treatment delivery remained the only statistically significant variable associated with treatment response. Notably, per protocol treatment delivery reflects both procedural and behavioral factors, including adequate lead placement to achieve stimulation coverage of the painful area and patient adherence to the prescribed daily usage. Nevertheless, recent findings from Odonkor et al. suggest that baseline psychosocial and behavioral factors, including physical activity, pain self-efficacy, and pain catastrophizing, may influence response to 60-day PNS [23]. In a second analysis, Odonkor et al. [24] developed nomogram models incorporating these psychosocial factors alongside clinical characteristics to forecast long-term response after 60-day PNS. Several variables explored by Odonkor et al. were not collected in the present study and therefore could not be assessed. Future prospective studies could complement these findings and further inform clinical practice.

In this per protocol analysis, post-hoc assessments suggest 60-day PNS can be effective across exploratory cohorts of common CLBP pain features, including the two most common types of axial CLBP: facetogenic and discovertebral (including discogenic) pain [11,12]. A majority of participants with history, physical exam, radiographic, and diagnostic block features consistent with facetogenic or discovertebral pain reported substantial relief with 60-day PNS. Facetogenic pain, traditionally treated with other interventional procedures (e.g., injections, RFA) that target the lumbar medial branches innervating the facet joints, appeared to respond well to treatment with 60-day PNS stimulating the lumbar medial branches. Findings from this study suggest that a positive response to a MBB was not statistically associated with achieving ≥50% pain relief, suggesting a positive MBB is not necessary to identify candidates that are likely to benefit from 60-day PNS for CLBP, as nearly half (49%) of participants who achieved meaningful relief did not have a positive response to an MBB (Table 4, Key Insight #2). Recent studies have explored the prognostic value of diagnostic nerve blocks for PNS outcomes. While Hoffmann et al. reported that relief from a pre-implant diagnostic peripheral nerve block was associated with the magnitude of pain relief at 3 months following temporary PNS implantation, there was no association at 6 months [25]. Similarly, D'Souza et al. reported no association between response to a diagnostic block and the magnitude of pain relief with PNS at 6 and 12 months [26]. Notably, both analyses pooled multiple nerve targets, whereas the present study evaluated a single diagnostic block of the lumbar medial branches. The relevance of diagnostic blocks for PNS outcomes may be limited, as 60-day PNS is thought to engage central mechanisms by generating comfortable afferent activity rather than via peripheral blockade (e.g., with ablation or anesthetic).

For exploratory cohorts with facetogenic and discovertebral pain features, reductions in pain with 60-day PNS were accompanied by reductions in pain interference and disability, with benefits sustained through 6 months (Table 4, Key Insight #3; follow-up beyond 6 months is ongoing). The positive outcomes observed across these exploratory cohorts may reflect the 60-day PNS treatment's proposed mechanism of action in addressing underlying central pain processing. Corroborated by multiple RCTs treating other painful regions, 60-day PNS is theorized to selectively activate large-diameter nerve fibers in the periphery to modulate central sensitization and disrupt the centrally maintained cycle of chronic pain [14], which may help explain the ability of percutaneous 60-day PNS to provide relief in patients with facetogenic and discovertebral pain features.

This post-hoc per protocol analysis has several limitations. The assessed variables were not controlled for outside of the eligibility criteria and therefore were assessed as observed. The absence of significant associations between assessed baseline characteristics and treatment response may reflect both the limited number and type of variables available in this study (since this study was not designed with this analysis as its primary objective), and the restricted variability of certain measures within the study's eligibility criteria. These findings do not preclude the existence of meaningful treatment predictors that were not captured in this dataset. Additionally, physical exam assessment criteria (e.g.*,* pain during forward flexion) were evaluated by multiple independent investigators, which could add variability and influence results. Medial branch blocks were evaluated using a single block with an 80% pain relief threshold as the success criterion. Dual diagnostic blocks are required in the majority of the U.S. before proceeding to RFA, though this is not the case worldwide. Of note, there remains ambiguity in clinical practice regarding the optimal number of blocks and the required level of pain relief to indicate a positive response before a patient is considered to be an appropriate candidate for RFA [27]. Additionally, a subset of participants (n = 8) had MBBs performed at outside facilities prior to enrollment. These pre-enrollment MBBs may have differed from the standardized MBB procedures within the study protocol with respect to technique, injectate, response criteria, or method of pain relief assessment. Pooling these with the 83 study-standardized MBBs introduces measurement heterogeneity that may impact the interpretability of MBB-related findings. While individual diagnostic criteria have known limitations, including limited predictive value of exam maneuvers [27], false-positive single anesthetic blocks [28], and the high prevalence of disc abnormalities in asymptomatic individuals [29], the combination of history, physical exam, imaging, and diagnostic block findings provides meaningful clinical context. This post-hoc per protocol analysis reports on trends and data as observed from a single group in this RCT; thus, the present analysis does not establish definitive causal relationships and results may be influenced by confounding factors not accounted for in the model. As a post-hoc per protocol analysis, the results may reflect those of a more favorable subset of participants who tolerated and adhered to the treatment as prescribed. Loss of randomization balance and the potential for selection bias are inherent limitations of per protocol analyses, and the results should be interpreted accordingly. In this context, this analysis provides practical clinical insight into how treatment delivery influences treatment outcomes with 60-day PNS.

### 4.1 Conclusions

Building on the findings of the RESET Clinical Trial, this post-hoc per protocol analysis primarily explored the relationship between treatment delivery and outcomes with 60-day PNS. As a secondary objective, this analysis also explored whether clinical variables were associated with treatment response within the study population defined by the trial's inclusion and exclusion criteria. This analysis found that per protocol treatment delivery of 60-day PNS (bilateral stimulation that generates sensations covering the majority of the painful region in the low back for 6-12 h per day) was significantly associated with achieving meaningful pain relief, as those participants had 3.04 times higher odds of achieving ≥50% reduction in average pain at 3 months. Clinical variables, including response to a diagnostic medial branch block, did not significantly impact outcomes among the population enrolled in this study. In an exploratory cohort analysis, percutaneous 60-day PNS demonstrated effectiveness among patients with history, physical exam, radiographic, and diagnostic block features consistent with facetogenic or discovertebral (including discogenic) pain. Reductions in pain were accompanied by meaningful improvements in pain interference and disability, with benefits sustained at all completed timepoints through 6 months. Together, this post-hoc analysis suggests that treatment delivery is an important determinant of achieving adequate relief with 60-day PNS, highlighting the importance of proper lead implantation, patient education, and treatment adherence in clinical practice.

## Ethics/ethical approval

This study received initial institutional review board (IRB) approval from a central IRB (Western IRB, now WCG IRB, Princeton, NJ; ID: 20192897) on December 30, 2019, and was registered on ClinicalTrials.gov/study/NCT04246281 on January 29, 2020. Twenty-one academic and private practice sites from all regions of the United States participated. All methods and procedures followed the principles of the Declaration of Helsinki. All participants provided written, informed consent prior to study procedures.

## Funding

The vast majority of funding for this study was made through a research grant from the Department of Defense (DoD) through the Office of the Assistant Secretary of Defense for Health Affairs via Award No. W81XWH-18-1-0800. Opinions, interpretations, conclusions and recommendations are those of the authors and are not necessarily endorsed by the DoD. The U.S. Army Medical Research Acquisition Activity, 820 Chandler Street, Fort Detrick MD 21702-5014 is the awarding and administering acquisition office. Additional funding needed to complete the study was provided by the study's sponsor, SPR. All data collection was conducted by independent academic and independent private practice research centers and all statistical analyses were performed by an independent, third-party biostatistics group (WCG Statistics Collaborative, Washington, DC).

## Conflicts of interest

S. Li, Z. McCormick, D. Lester, M. DePalma, C. Gilmore, J. Jameson, M. Desai, T. Weaver, N. Lad, S. Davidoff, D. Trainor, K. Amirdelfan, M. Engle, T. Deer, T. Lee, F. Vetri, M. Bindal, M. Tornero-Bold, and M. Nasseri reports financial support was provided by US Department of Defense. S. Li, Z. McCormick, D. Lester, M. DePalma, C. Gilmore, J. Jameson, M. Desai, T. Weaver, N. Lad, S. Davidoff, D. Trainor, K. Amirdelfan, M. Engle, T. Deer, T. Lee, F. Vetri, M. Bindal, M. Tornero-Bold, and M. Nasseri reports financial support was provided by SPR. S. Li, C. Gilmore, J. Jameson, M. Desai, T. Deer, and S. Cohen reports a relationship with SPR that includes: consulting or advisory. T. Deer, M. Desai, W. Clark, M. McGee, and J. Boggs reports a relationship with SPR that includes: equity or stocks. W. Clark, M. McGee, and J. Boggs reports a relationship with SPR that includes: employment. Given Z. McCormick's and S. Cohen's roles as Executive Editors at Interventional Pain Medicine, Z. McCormick and S. Cohen had no involvement in the peer review of this article and had no access to information regarding its peer review. Full responsibility for the editorial process for this article was delegated to another journal editor. If there are other authors, they declare that they have no known competing financial interests or personal relationships that could have appeared to influence the work reported in this paper.

## Data Availability

The datasets generated during and/or analyzed during the current study are available from the corresponding author on reasonable request.

## APPENDIX 1 **Study Eligibility Criteria**

### Inclusion Criteria

• Age ≥21 years and ≤75 years
• Able to understand and comply with study requirements and provide written informed consent
• Chronic low back pain (*i.e.,* pain lasting longer than 6 months, where the vast majority and center of pain is in the lumbar region, L1-L5 vertebral levels)
• Reports an average low back pain score ≥4 on a scale of 0-10 (BPI5)
• Previous use of at least two types of LBP therapies (e.g., medications, physical therapy, injections, etc.)
• At least 4 weeks of stable pain treatment or medication as indicated by subject reported medication history (*i.e.,* no new treatments or change in medication for pain in the last 4 weeks)
• Reports active health insurance coverage (e.g.*,* through commercial insurance, Medicare, Medicaid, Tricare)

### Exclusion Criteria

• Radicular leg pain (e.g.*,* pain that spreads to the lower extremities) or referred pain outside the lumbar region (e.g.*,* sacral pain, hip pain) that is ≥ 4 on a scale of 0-10, BPI5
• Pain in the thoracic or cervical region that is ≥ 4 on a scale of 0-10, BPI5
• Pain from the sacroiliac joint, as determined by the Investigator
• Signs of infection on or around the low back, or other conditions that increase risk to the subject in the opinion of the Investigator (e.g.*,* valvular heart disease that creates an infection risk, compromised immune system, history of recurrent skin infections)
• Signs of serious underlying cause of low back pain as determined by the Investigator, (e.g.*,* cancer, chronic infection, referred visceral pain, metabolic bone disorder)
• Deep brain stimulation (DBS) system, an implanted active cardiac implant, or any other implantable neurostimulator whose stimulus current pathway may overlap the Percutaneous 60-Day PNS stimulator's current pathway
• Anesthetic or corticosteroid injections in the low back (including trigger point, epidural, intrathecal, facet, or sacroiliac joint injections) within the last 3 months (not including a diagnostic medial branch block), or botulinum toxin (Botox) injection in the low back within the last 6 months
• Prior radiofrequency ablation of the lumbar medial branches (L1-L5) within the last 6 months
• Prior lumbar surgery
• Clinically relevant stenosis of the central canal or foramina as determined by the Investigator (e.g.*,* symptoms of neurogenic claudication)
• Greater than mild lumbar scoliosis (*i.e.,* >20°)
• Condition that could impact response to percutaneous PNS (e.g.*,* fibromyalgia confirmed by a specialist, multiple sclerosis, spinal cord injury)
• History of significant trauma to the lumbar spine or paraspinal musculature (e.g.*,* burst fractures or fracture dislocations)
• Score of >20 on the Beck Depression Inventory (BDI-II)
• Average Pain Interference score <4 using BPI9
• Obese with a body mass index (BMI) > 40
• Current illicit substance abuse or high-dose opioid dependence (e.g., daily opioid usage ≥90 mg morphine equivalents)
• Current participation, or less than 30 days from completing any drug or device trial, or previously received Percutaneous 60-Day PNS for LBP
• Pending litigation, workers compensation or other secondary gain issues
• Tape or adhesive allergy
• Allergy to all local anesthetic agents (e.g.*,* lidocaine)
• Any other medical condition that may interfere with ability to participate in a clinical trial as determined by the Investigator (e.g.*,* bipolar disorder)
• Vulnerable populations (e.g.*,* prisoners, individuals that report to investigators)
• Bleeding disorder (e.g., hemophilia)
  o Note: anticoagulant use is not considered a bleeding disorder.

### Additional Inclusion Criteria *(assessed before randomization)*

• Average pain intensity score of ≥4 (determined by calculating the mean “average pain” collected in a 7-day baseline diary, using Question #5 on the BPI-SF)

### Additional Exclusion Criteria *(assessed before start of treatment with Percutaneous* 60-Day *PNS)*

• Pregnancy (Group #1 only)
